# Predicting the number of defects in a new software version

**DOI:** 10.1371/journal.pone.0229131

**Published:** 2020-03-18

**Authors:** Ebubeogu Amarachukwu Felix, Sai Peck Lee

**Affiliations:** Faculty of Computer Science and Information Technology, University of Malaya, Kuala Lumpur, Malaysia; Universiti Sains Malaysia, MALAYSIA

## Abstract

Predicting the number of defects in software at the method level is important. However, little or no research has focused on method-level defect prediction. Therefore, considerable efforts are still required to demonstrate how method-level defect prediction can be achieved for a new software version. In the current study, we present an analysis of the relevant information obtained from the current version of a software product to construct regression models to predict the estimated number of defects in a new version using the variables of defect density, defect velocity and defect introduction time, which show considerable correlation with the number of method-level defects. These variables also show a mathematical relationship between defect density and defect acceleration at the method level, further indicating that the increase in the number of defects and the defect density are functions of the defect acceleration. We report an experiment conducted on the Finding Faults Using Ensemble Learners (ELFF) open-source Java projects, which contain 289,132 methods. The results show correlation coefficients of 60% for the defect density, -4% for the defect introduction time, and 93% for the defect velocity. These findings indicate that the average defect velocity shows a firm and considerable correlation with the number of defects at the method level. The proposed approach also motivates an investigation and comparison of the average performances of classifiers before and after method-level data preprocessing and of the level of entropy in the datasets.

## 1 Introduction

Software testing requires considerable time and consumes almost half of the entire budget of a project, thus making it an expensive phase of the software development life cycle (SDLC) [1], [2]. Therefore, a reliable test plan must consider the available resources while reducing the number of defects in a program, thus lessening the workloads of both managers and the testing team. Method-level defect prediction can help to ensure that software testing receives the necessary attention within the software engineering community. To this end, it is necessary to provide advance information (i.e., prior information about the future) on new versions of software. Existing studies have mainly focused on class-level binary classification, with the objective of improving classifier performance. However, little or no research has been directed towards estimating the number of defects in a new version of software at the method level. Prior to the current study, the author of [3] reported that certain variables can be derived to estimate the future number of defects in a new version of software at the class level. The current study extends these findings to ascertain their applicability at the method level.

The ability to estimate the numbers of software defects at both the class and method levels in advance can greatly assist software teams in ensuring the reliability of a program throughout the SDLC and can also aid in decision-making. Thus, further research is required to investigate whether certain derived optimal variables, such as defect density, defect velocity and defect introduction time, have some correlation with the number of defects and hence are applicable in building models for defect prediction. In previous work [3], we reported that through the relationship of these optimal variables with the current number of defects in a software program, the number of defects in a future version of that program can be predicted at the class level by using the average defect velocity obtained for the current version to construct regression models. Here, we further investigate these findings to verify whether the proposed approach can also be applied at the method level. We believe that in the end, such a prediction model will provide invaluable support and guidance during software testing [4]. Hence, there is a need for a feasible test blueprint in software engineering, driven by the importance of properly using the available resources during software testing and of delivering quality software products to users at all times.

Identifying and fixing software faults at the method level can be a cost-intensive prospect if such remediation is executed only after the delivery of the software product to the end user. This is because there are always greater risks involved in identifying the cause of errors after a product has gone into operation, and the cost of correcting such errors is usually high, from the perspective of either the end user or the developers [5]. End users, developers and other software stakeholders must all expend resources to fix the damage caused by defects in software products. To avoid such an undesirable situation, it is important to estimate the number of software defects at the method level in advance to possibly prevent such faults from occurring.

In this study, three different phases of experiments were carried out. The first experimental phase dealt with method-level data preprocessing evaluated via binary classification, the second phase addressed regression analysis with the goal of determining the number of method-level defects in a new product version, and the third investigated correlations to determine the impacts of the derived variables on the number of defects at the method level. An experiment was carried out on the Finding Faults Using Ensemble Learners (ELFF) open-source Java projects publicly available at [6], which contain 289,132 methods. At the method level, the results show correlation coefficients of 60% for the defect density, -4% for the defect introduction time, and 93% for the defect velocity. Furthermore, the results for the predicted numbers of defects likely to be present in the future versions of the ELFF projects were compared with the current numbers of defects.

**Fig 1:**
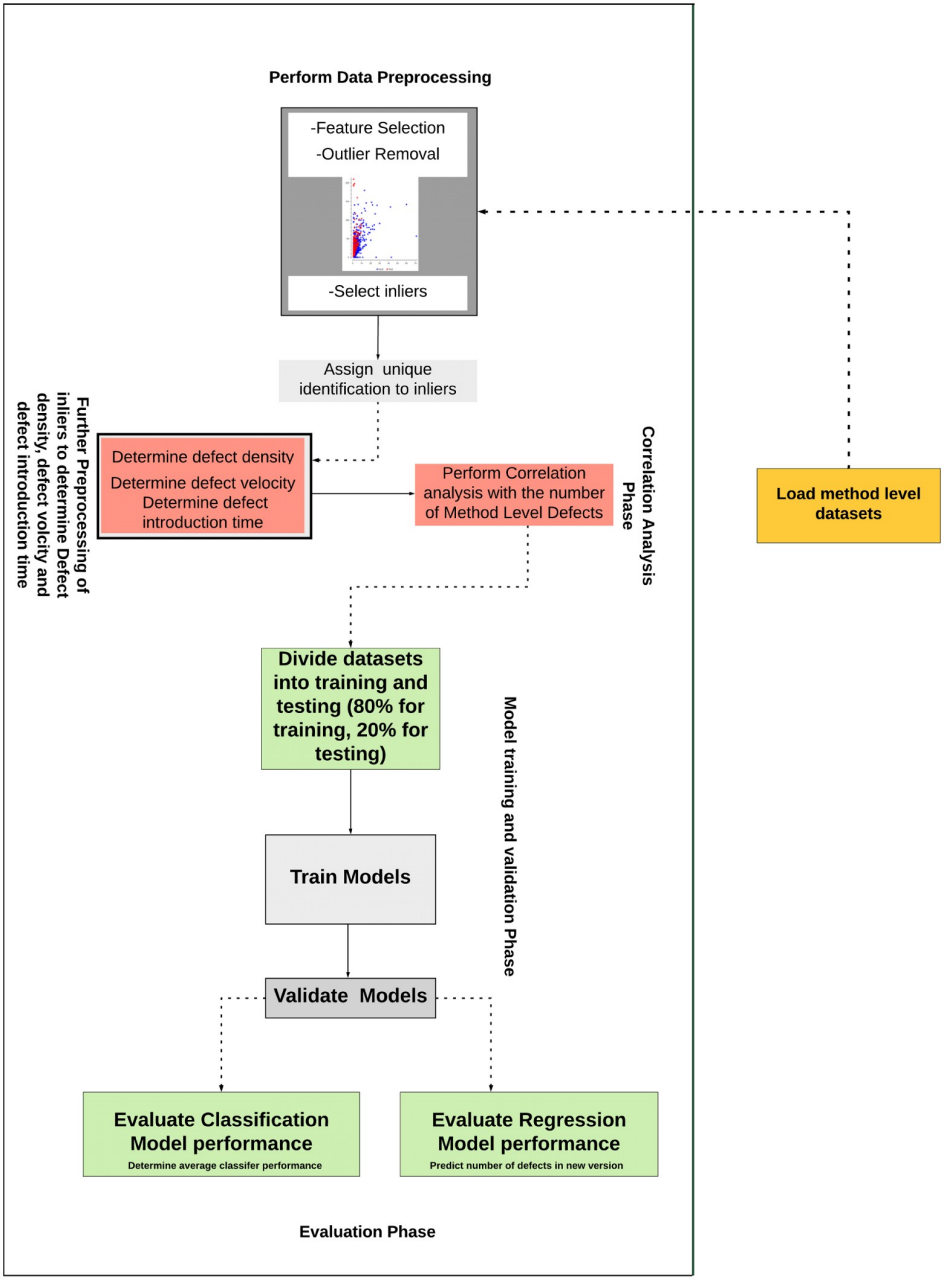
Overview of the prediction framework.

Another contribution of this study is the application of an optimal approach to ensure that the datasets used were properly preprocessed despite the uncertainties associated with the existing datasets applied in machine learning studies. Hence, we also present the results obtained when various learning algorithms were applied to both the raw and preprocessed datasets. In addition, to properly assess the uncertainties associated with imbalanced data in terms of information entropy, we compute the results obtained for the learning algorithms when applied to the method-level ELFF datasets. The reason for this computation is to determine the levels of inconsistency in the datasets. Fig 1 presents a pictorial overview of the method-level defect prediction framework.

### 1.1 Overview of the prediction framework

To date, the underlying factors responsible for an increase in the number of method-level defects in a software product (for example, the defect acceleration, as characterized by the defect velocity and defect introduction time) have not been fully considered in defect prediction studies. In view of the fact that the existing literature has not demonstrated a way to bridge this gap, this manuscript therefore aims to address this need by presenting a cost-effective data preprocessing approach for enhancing the quality of the method-level data applied in defect prediction studies while revealing the factors that contribute to an increase in the number of defects in a software product. Thereafter, it is demonstrated how the relationship between the defect acceleration and the number of defects can be useful in predicting the number of defects in a new software version. Fig 1 presents the step-by-step approach, from preprocessing the method-level datasets to predicting the number of defects in a new software version using the information obtained from the current software version. In addition, the preprocessed datasets are applied in evaluating the average classifier performance. The preprocessing of a method-level dataset is performed as follows. First, relevant features are selected from the dataset. The reason for such feature selection is to ensure that only meaningful attributes that show sufficient correlation with the target class (defective class) are selected. Redundant and irrelevant features are discarded. Second, outlier removal is performed. Outliers are data points that lie far away from the main cluster(s) of data. Outliers may occur in datasets as a result of measurement variations or may be an indication of experimental error. In this study, outliers were identified and removed during the preprocessing phase because they might impact model performance. The preprocessing time for the method-level datasets applied in the current study is also presented. Following outlier removal, the resulting inliers are further preprocessed by determining the average defect density, average defect velocity and average defect introduction time. Furthermore, the correlations of the average defect density, average defect velocity and average defect introduction time with the number of defects are determined. The preprocessed datasets are divided at a ratio of 80% to 20% for training and testing the defect prediction models, respectively. The reason for training the models with 80% of the data is to ensure that the models learn accurately and independently via cross-validation sampling to avoid bias. The models are then validated with the remaining 20% of the data for predicting the number of defects in a new software version, and the average classifier performance is also determined on the test data. In the current manuscript, we demonstrate how imbalanced datasets can be preprocessed to avoid bias by applying the proposed data preprocessing framework. In addition, the time required to preprocess the method-level datasets is presented. This manuscript also shows how certain variables such as the average defect velocity can be applied in predicting the number of defects at the method level in a new software version. Furthermore, this manuscript demonstrates the need to determine the average performance of classifiers when applied to imbalanced data as well the average information entropy in classification algorithms.

The remainder of this paper is organized as follows. Section 2 presents related work. Section 3 describes the materials and methods. Section 4 presents our experimental methodology and outcomes. Section 5 presents our results and discussion. In Section 6, we present our conclusion and directions for future work.

## 2 Related work

Researchers have made great efforts to develop various techniques to envision the number of defects in software at the code level [7]. In addition, several studies have proposed approaches for improving the outcome of software defect prediction with a focus on binary classification, for example, [8], [9], [10], [11], [12], [13], [14], [15], [16], [17]. These studies have focused on improving the performance of learning algorithms. For instance, while investigating methods of improving the classification accuracy of learning algorithms, Galar et al. [18] and He [19] reported that the performances of these algorithms can be improved by considering combinations of individual metrics when confronting challenges related to imbalanced data. Lessmann et al. [15] evaluated the performance superiority of one classifier over another. Using an alternative improvement approach, Taba et al. [20] investigated how to increase the performance of a bug prediction model by means of metric-based antipatterns. Batista et al. [21] performed an extensive experimental assessment of the performances of learning algorithms when applied to imbalanced data and reported that class imbalance does not completely prevent the successful application of learning algorithms. Notably, such learning algorithms are capable of classifying a module as either defective or defect-free. Petrić [7] reported that the current prediction models handle defect prediction in a black-box manner. This is a weakness of the existing prediction models since they do not enable the prediction of the number of defects but rather focus on classification, which is merely an attempt to forecast whether a software program will be defective [22].

On the other hand, the application of regression models can allow a software team to determine which models can best reveal the relationship between the independent and dependent variables [7]. While attempting to address the lack of approaches for anticipating the number of future defects in a software program, Bernstein et al. [23] noted that if the number of defects in a new version of software can be predicted, both software managers and other stakeholders will benefit.

Nagappan and Ball [24] presented a technique using a set of relative code churn measures for the early prediction of the software defect density. Their results showed that absolute measures of code churn are poor predictors of defect density. Meanwhile, Bernstein et al. [23] extracted a certain number of temporal features, such as the number of revisions and the number of reported issues, from the CVS and Bugzilla repositories to predict the number of defects for the Eclipse project. Their results indicate that by using certain temporal features, a prediction model can be developed to predict whether a source file will contain a defect. That study achieved noteworthy results in predicting the number of software defects, although it was limited to only six Eclipse plugins, representing a single project.

To enable the estimation of the number of defects in a new software version, our previous work [3] proposed certain prediction models constructed using the code design complexity, defect density, defect introduction time and defect velocity. The results obtained suggest that the number of defects in a new release can be achieved at the class level of the software. Beyond the work reported in [3], none of the existing studies has considered the prediction of the numbers of software defects at both the class and method levels using the aforementioned variables. Hence, the current study aims to extend this approach based on derived variables to forecast the number of defects expected to be present in a future software version at the method level. One of the drawbacks of the previous study is that the proposed approach was not applied at the method level; in addition, that study did not report detailed information on the regression models, such as percentage errors. Based on the above concerns, we can conclude that the quality of the data applied in that study was low. If the size of a software program can be predicted, as reported by Laranjeira [25] and Dolado [26], then a method of predicting the possible number of defects likely to be present in a future release at the method level is also needed. Hence, the current study attempts to provide such an approach. Therefore, to fill this gap in the literature regarding method-level defect prediction, the current study presents a detailed analysis of the approach applied and clear evidence of reliable datasets. Above all, the accuracy of these prediction models depends on the quality of the datasets used, which determines whether the learning algorithms applied to these data can learn accurately. In addition, sufficient dataset quality will enable these learning algorithms to gain and transfer knowledge within and across projects. Notably, the quality of the information gained from a reliable data source can enhance the intertask learning mechanism, as reported by the authors of [27]. Furthermore, the authors of [28] confirmed that datasets are imbalanced and inaccurate by nature and consequently must be freed from bias before any learning algorithm can be applied. By the same token, if a model is trained with a reliable dataset, this can increase the efficiency of the model as well as optimizing its output [29]. Therefore, there is a need to devise an accurate means of acquiring reliable datasets suitable for training prediction models to achieve high performance [28]. Therefore, to ensure that the datasets applied in prediction studies are of high quality, this manuscript presents a step-by-step data preprocessing approach to ensure that these datasets are free from bias before the application of learning algorithms to avoid misleading results.

## 3 Materials and methods

The aims of this study are to extend the idea previously reported in [3] to demonstrate how certain optimal variables can be applied both in data preprocessing and in predicting the possible number of defects in a future version of a software product using information available from the current version at the method level of the software. The term **optimal variables**, as applied in this paper, refers to the defect density, defect velocity and defect introduction time determined through decisions made in every stage of data preprocessing so as to obtain an optimal result. In our previous work [3], we have demonstrated that such an approach is achievable at the software class level. Therefore, the current study further investigates these findings to ascertain the possibility of applying the proposed approach at the software method level. We believe that if such an approach is achievable at the method level, it will provide the software team with additional information on the possible defects in a future version and also assist in decision-making. In addition, we hope to compare the results obtained when various learning algorithms are applied to unprocessed data with the results obtained on preprocessed data and determine whether the imbalance in the data has an adverse effect on the final learning outcome at the method level. To satisfy the objectives of the current study and properly investigate the findings reported in [3], several research questions are formulated.

### 3.1 Formulation of research questions

This subsection presents the research questions addressed in this study. One of the objectives of this study is to determine how the techniques proposed in our previous work [3] can be applied in data preprocessing to facilitate the estimation of the number of method-level defects. Thus, the research questions (**RQs**) addressed in our study are as follows:

RQ1 How many studies have applied the aforementioned optimal variables when performing data preprocessing for defect prediction at the method level?
RQ2 How do these variables address method-level data quality issues?
RQ3 What are the limitations of the current research in the supervised machine learning domain?

To address these research questions, we considered several factors. For instance, to address **RQ1**, we first investigated articles in specific topic areas related to defect prediction to determine whether these optimal variables have been applied in predicting the possible number of defects in a future software version at the method level. To address **RQ2**, we focused on the results of the individual studies to determine whether these variables are capable of addressing specific data-related issues. For **RQ3**, which concerns the limitations of the current state of research in this area, we considered one specific question:

**RQ3.1**. Is there evidence that these optimal variables have not been fully applied in machine learning studies when attempting to predict the number of defects in a new version of software at the method level?

### 3.2 Definitions of metrics

Defect density is an essential attribute for determining software reliability; a software product can be released on the market only after its defect density is considered low enough to avoid criticism [30]. The quality of a software product can be evaluated based on its defect density [31], [32]. A software product with a high defect density tends to perform less well, while a product with a low defect density has the ability to run without failure [33], [34]. Specifically, a high defect density indicates that a software program contains a large number of defects; as such, the ability to estimate defect density makes it easier for a testing team to focus on identifying and correcting as many defects as possible with limited resources [33]. Software companies tend to benefit from the early detection of defects that are likely to be present in software, which is facilitated by knowledge of the defect density in a software product of interest [24], [35], [36]. In addition, defect density data provide information that can help development teams keep proper records while attempting to reduce the number of defects in upcoming versions of a current product [33].

Moreover, defect density also provides the development team with helpful information for keeping track of the progress made in reducing the number of defects during software project transitions [33]. It is also important to note that while overall software quality is a matter of concern, the aspects that require the greatest attention are mainly the components with the highest defect densities; thus, the ability to determine defect density is key [37]. Furthermore, based on the information provided by the defect density of a software product, the number of defects in an upcoming release of that product can be estimated [37]. Based on the relevant mathematical relationship, the optimal variables chosen as the basis of the current study are derived to provide a means of determining the possible numbers of defects likely to be present in various software products, as reported in [3]. If the number of defects can be determined prior to software testing, it will be easier for the testing team to focus on addressing as many defects as possible with the limited resources available [33]. Typically, throughout the SDLC, software developers aim to meet the project deadline and thus carry out software development as quickly as possible, without proper elaboration of the impact of such speedy transitions. Such high-velocity transitions through the SDLC can expose a software product to defects. Therefore, to improve software quality, it is wise to determine the impact of the average defect velocity on the number of software defects as a software product transitions from one phase of the SDLC to another. Any significant improvement in the SDLC will lead to a reduction in the rate at which defects occur, reduce the need for software rework and ultimately improve software quality and productivity [38]. Hence, it would be in the best interests of the machine learning community if the number of defects in a new version of software could be successfully estimated. In addition, it would be beneficial to have an idea of the rate at which these defects occur and the impact of this rate on software products. In this study, we hypothesized that there is a possibility that the proposed optimal variables have an impact on the number of defects at the software method level since the defect velocity has a strong impact at the class level, as reported in [3]. Further hypotheses were also formulated in the current study. To confirm these hypotheses, an experiment was carried out in which the defect velocity was used to predict the numbers of defects in software programs at the method level, with the goal of assisting software teams in improving the quality of software through proper management. The defect velocity was chosen because it exhibits a higher correlation with the number of defects than either the defect density or defect introduction time does. Notably, a well-managed software development process will result in less defective software by allowing the speed of software development to be increased while ensuring that the demands of the customers are met [39].

**Fig 2:**
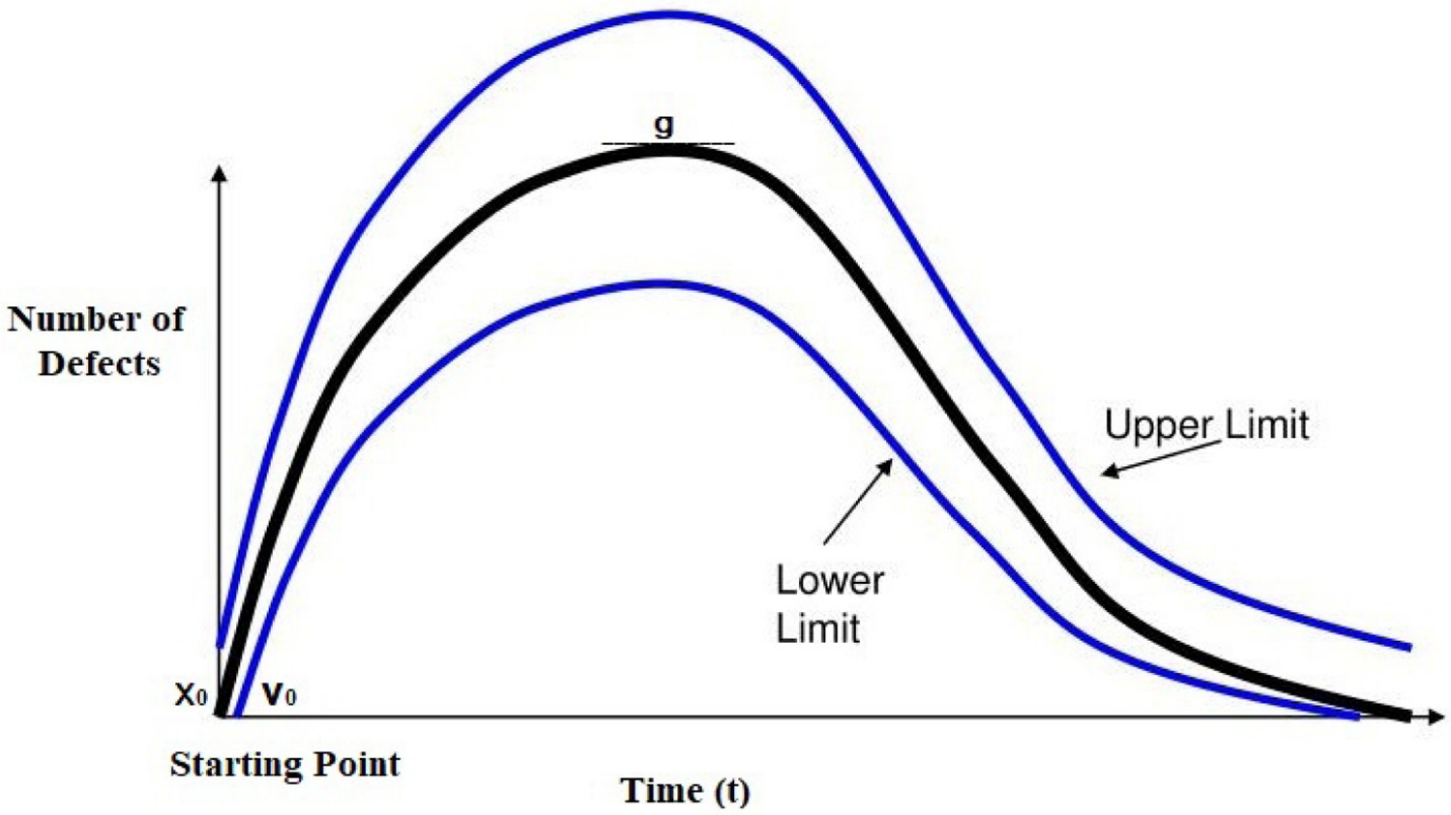
Rayleigh distribution curve [40].

At the starting point of a project, the number of defects is zero, as illustrated in Fig 2. The chance of defect introduction increases over time as the project proceeds from one phase to the next. With further phase transitions during software development, the defect acceleration increases, which can lead to an increase in the number of defects. The defect acceleration is the change in the defect velocity at a given instant of time. The increase in the number of defects and the defect density are therefore functions of the defect acceleration.

If the defect acceleration is low, then the number of defects will also be low, and because the defect acceleration is defined as the change in the defect velocity over time, the defect density, which is affected by the defect velocity, will be low as well. If the defect acceleration increases, this increased acceleration will lead to an increase in the number of defects and, hence, an increase in the defect density; therefore, the defect density *g* is a function of the defect acceleration, which characterizes defect occurrence given a constant project size. The defect acceleration can be calculated as the change in the defect velocity over the change in time, as follows:

$$defect\;acceleration=\frac{\Delta velocity(v)}{\Delta time(t)}$$ (1)

**Defect density**: The defect density *g* represents the ratio of the number of defects to the size of a software project. An increase in the number of defects in a software project will result in an increase in the defect density [3]. The defect density as applied in this study is measured in number per unit project size and is calculated using the following equation:

$$defect\;density=\frac{no.of\;defects}{project\;size}$$ (2)

**Defect velocity**: The defect velocity *v* is the change in the defect position with respect to time *t* and is measured in units of defects per day. The defect position here ranges from the requirement phase of the SDLC to the implementation phase and indicates when defects might have occurred during software development.

**Defect introduction time**: The defect introduction time *t* is the time at which defects occur in a software product and is measured in days.

#### 3.2.1 Hypotheses

To achieve the objectives of the current study, the following hypotheses were formulated:

(a) The defect density at the method level in software is influenced by the number of defects.
(b) The increase in the number of defects present at the method level depends on the rate at which defects occur at the method level.
(c) An increase in the rate at which defects occur will lead to an increase in the defect density.
(d) At a constant project size, the defect density at the method level is equivalent to the defect velocity divided by the defect introduction time. Thus,
  $$\frac{no.of\;defects}{project\;size}∼\frac{defect\;velocity(v)}{defect\;introduction\;time(t)}$$ (3)
(e) The reliability of software is a function the number of defects in that software.

### 3.3 Machine learning models for data analysis

As mentioned earlier, one of the objectives of the current study is to extend the implementation of the previously proposed optimal variables to the preprocessing of method-level datasets and predicting the number of defects in a new version of software. Furthermore, we compare the results obtained when various learning algorithms are applied to unprocessed data with the results obtained on preprocessed data. To achieve this objective, both classification algorithms and evaluation metrics are required. For this purpose, in the current study, we have implemented several classifiers, i.e., machine learning models for data analysis tasks. The list of classifiers applied in the current study includes naïve Bayes, logistic regression (LR), neural network, K-nearest neighbors (KNN), support vector machine (SVM) and random forest (RF) classifiers.

**Naïve Bayes**: A naïve Bayes classifier is an effective tool that is capable of generating accurate prediction results, and it is a suitable choice for classification problems involving variates of independent variables [41]. It is capable of handling large datasets and often outperforms more sophisticated classification models. The naïve Bayes model is derived from Bayes’ theorem under the hypothesis of independence among the variables. Bayes’ theorem provides an avenue for estimating the conditional probability *prb*(*a*|*b*) from *prb*(*a*), *prb*(*b*) and *prb*(*b*|*a*). For a large dataset, the posterior probability of class *a* is calculated as

$$prb\left(a|b\right)\propto prb(b_{1}|a)prb(b_{2}|a)\ldots prb\left(b_{n}|a\right)prb\left(a\right)$$ (4)

where *prb*(*a*|*b*) = the posterior probability of class *a* given attribute *b*,

*prb*(*a*) = the prior probability of class *a*,

*prb*(*b*) = the prior probability of attribute *b*,

*n* = the number of instances, and

*prb*(*b*|*a*) = the likelihood, that is, the probability, of attribute *b* given class *a*. The class with the highest posterior probability is the predicted outcome.

**Logistic regression (LR)**: LR estimates the likelihood of an action that has two possible values (binary classification) and thus is suitable for a predictive analysis conducted when the possible values of the independent variables are 0 and 1. The LR model is employed to analyze data and to interpret the relationship between one dependent binary variable and one or more independent variables.

**Neural network**: A neural network classifier is a classification model inspired by the neurons in the human brain. The units in a neural network perform tasks similar to those of human neurons. Such a network has an input layer, an output layer, and one or more hidden layers in between. During classification, data pass through the units of the neural network, which perform various mathematical computations. These units interact with each other via connections linking the various layers. Each connection has a number, called a weight, associated with it. When the neural network receives an input, it processes that input by performing calculations based on the connection weights to produce an output. A neural network is trained by adjusting the values of the connection weights; this is achieved by means of a backpropagation algorithm, which trains from the input layer to the output layer and vice versa. Furthermore, a neural network can be trained by reducing the value of a loss function over a training set using a gradient-based method [42].

**K-nearest neighbors (KNN)**: A KNN classifier is a straightforward classification model that considers all possible outcomes and classifies an instance in advance based on a resemblance factor. An instance is classified based on the voting strength of its neighbors. In the classification result, each instance is assigned to the class with the highest number of corresponding votes, as determined by a distance factor. For the distance factor in this paper, we use the Euclidean distance between two neighbors *a* and *b*, which also represents the length of ($\bar{ab}$). In Cartesian coordinates, if the coordinates of two different points in a Euclidean k-space are given by *a* = (*a*_1_, *a*_2_, …, *a*_*n*_) and *b* = (*b*_1_, *b*_2_, …, *b*_*n*_), then the distance *d* from point *a* to point *b* (or from *b* to *a*) is determined by the Pythagorean formula:

$$d\left(a,b\right)=d\left(b,a\right)=\sqrt{{\left(b_{1}-a_{1}\right)}^{2}+{\left(b_{2}-a_{2}\right)}^{2}+\ldots +{\left(b_{n}-a_{n}\right)}^{2}}$$ (5)

$$d\left(a,b\right)=d\left(b,a\right)=\sqrt{\sum_{i=1}^{n}{\left(b_{i}-a_{i}\right)}^{2}}$$ (6)

**Support vector machine (SVM)**: An SVM classifier finds the line of best fit that maximizes the margin between two classes that are linearly separable. The SVM classifier treats every instance of a class as a vector of the corresponding input variables. Thus,

$$v=(x_{1},x_{2},\ldots ,x_{n})$$ (7)

Consequently, a dataset *D* takes the form of pairs of vectors and classes:

$$D=\left\{(x_{1},y_{1}\right),\left(x_{2},y_{2}\right),\ldots ,x_{n},y_{n})\}$$ (8)

where *x* = the vectors, *y* = the classes associated with the vectors (either +ve or -ve), and *n* = the number of instances.

Then, the SVM locates the two closest points between the two classes. The SVM connects these two points with a connection line and draws a perpendicular line bisecting this connection line. Thus, the two closest points define the line of best fit. This line of best fit has an intercept *b* and a normal vector $\vec{w}$ that represents a weight vector such that all point vectors $\vec{x}$ on the line satisfy the linear equation

$${\vec{w}}^{T}\vec{x}=-b$$ (9)

We can rewrite Eq (9) as

$${\vec{w}}^{T}\vec{x}+b=0$$ (10)

such that the scalar product of the weight vector and the point vector is equal to the intercept of the line of best fit. From Eq (8), the training dataset can be written as

$$D=\left\{({\vec{x}}_{i},y_{i})\right\}$$ (11)

in the form of point vectors $\vec{x_{i}}$ paired with their corresponding classes *y*_*i*_. Thus, the linear classification as a function of the point vector $\vec{x}$ becomes

$$f\left(\vec{x}\right)=sign\left({\vec{w}}^{T}\vec{x}+b\right)$$ (12)

**Random forest (RF)**: An RF classifier consists of a large collection of decision trees. These random trees are said to make up a forest. A reasonable number of decision trees are generated based on random selections of data and variables. The RF predicts the class of the dependent variable based on the number of trees. The decision trees are then used to predict the classification outcome. RF classifiers can sometimes outperform other sophisticated classifiers [43], [44].

These learning algorithms have different parameter settings, which we have maintained throughout the experiments. For instance, in the naïve Bayes classifier, the parameter for M-estimation was set to 2.0 with 100 sampling points. For the LR classifier, L2 regularization (square weights) was used, with a training error cost (*C*) of 100 and preprocessing based on a normal data distribution. The neural network classifier was configured with 20 hidden-layer neurons, a regularization parameter of 10 and a maximum of 300 iterations. For the KNN classifier, the number of neighbors was set to 5, and Euclidean normalization was applied to continuous attributes. For the SVM classifier, the objective function was *C*−*SVMCost*(*C*) = 1.00; the radial basis function (RBF) kernel was used, i.e., *exp*(−*γ*∥*X* − *X*′∥^2^), where ∥*X* − *X*′∥^2^ is the squared Euclidean distance between the feature vectors of two samples and *γ* = $\frac{1}{2\sigma^{2}}$, with *σ* being a free parameter; and the numerical tolerance was 0.0010, with estimated class probabilities and normalized data. For the RF classifier, the number of trees in the forest was 10, and the maximum number of nodes per tree was defined to be 5 as the criterion to stop splitting.

#### 3.3.1 Evaluation metrics applied in determining the average classifier performance

In this subsection, we present a list of the evaluation metrics applied in determining the performances of the classification models applied in this study. We applied a large number of metrics because the use of many evaluation metrics increases the likelihood of obtaining useful information on the model accuracy, such as the mean performance, and thus results in more reliable predictions [13]. The following notations are used in this subsection: TP = the number of instances that are actually positive and are also predicted to be positive, also called true positives; FP = the number of instances that are negative but are predicted to be positive, also called false positives; FN = the number of positive instances that are incorrectly predicted to be negative, also called false negatives; and TN = the number of negative instances that are correctly predicted to be negative, also called true negatives.

**Classification accuracy** (**CA**): The ratio of the number of correct predictions to the total number of predictions. The CA is calculated as follows:

$$Accuracy=\frac{TP+TN}{TP+TN+FP+FN}$$ (13)

**Precision**: The proportion of all predicted positive instances that are correctly predicted.

$$Precision=\frac{TP}{TP+FP}$$ (14)

**Recall** (**sensitivity**): The proportion of all actually positive instances that are correctly predicted.

$$Recall=\frac{TP}{TP+FN}$$ (15)

**Specificity**: The proportion of all actually negative instances that are correctly predicted.

$$Specificity=\frac{TN}{TN+FP}$$ (16)

**Matthews correlation coefficient** (**MCC**): Applied in binary classification as a measure of the achieved performance, the MCC is an important correlation coefficient that represents an overall assessment of the observed and predicted classes in binary classification. It considers all four aspects of the confusion matrix, including the true negatives [45]. It is calculated directly from the confusion matrix as follows:

$$MCC=\frac{TP\times TN-FP\times FN}{\sqrt{\left(TP+FP)(TP+FN)(TN+FP)(TN+FN\right)}}$$ (17)

**J-coefficient**: The sum of the recall and specificity minus one. It is equivalent to the recall minus the false positive rate (FPR).

J-coef=Recall-FPR=Recall+Specificity-1 (18)

**F-measure** (**F-score** or **F1**): The harmonic average of the precision and recall. It is calculated using the following formula:

F-score=2×(Precision×Recall)/(Precision+Recall) (19)

**Area under the ROC curve** (**AUC**): A metric based on a graphical representation of the performance of a binary classifier. It can also be used to compare different classifiers. A larger AUC value indicates better classifier performance. As reported in [46], the possible values of the AUC lie between 0 and 1. An AUC value of 0 indicates the worst possible performance, whereas a value of 1 indicates the best possible performance.

**Geometric mean** (**G-mean**): The square root of the product of the recall and precision, as proposed in [47] and [48]. Thus, the geometric mean is calculated as follows:

$$G-\text{mean}=\sqrt[{}]{Recall\times Precision}$$ (20)

Eq (20) represents the geometric mean for the minority target class (True class).

**Brier score** (**BS**): A score that measures the accuracy of probabilistic predictions. Tantithamthavorn et al. [46] applied the Brier score in their study to measure the gap between the estimated probability and the result achieved. A Brier score of 0 is the best achievable score, and 1 is the worst achievable score. The Brier score is calculated using the following formula:

$$BS=\frac{1}{N}\sum_{t=1}^{N}{\left(f_{t}-o_{t}\right)}^{2}$$ (21)

where *f*_*t*_ = the forecasted likelihood of occurrence,

*o*_*t*_ = the true event result at time *t*, and

*N* = the number of predicted instances.

**Information score (IS)**: The average amount of information per classified instance, as defined in [49].

The **average classifier performance (${\bar{P}}_{av}$)**, which represents the mean performance of a given classifier for the target class, is calculated as follows:

$${\bar{P}}_{av}=\frac{1}{n}\sum_{i=1}^{n}p_{i}=\frac{1}{n}(p_{1}+p_{2}+\ldots p_{n})$$ (22)

where *n* = the number of experiments performed,

${\bar{P}}_{av}$ = the average classifier performance, and

*p*_*i*_ = the individual performance in the *i*-th experiment (here, each experiment corresponds to a different project or dataset).

**Standard error** *ε*: In a regression analysis, there is the possibility of variations in measurements. The magnitudes of these variations are often high but sometimes low relative to the measurements themselves. The standard error can be used as an indicator of the reliability of sample estimates relative to the actual measurements. The standard error is applied to check the prediction accuracy in terms of the difference between the actual and predicted numbers of defects.

**Average information entropy**: In a situation where the average uncertainties of an information source regarding the different possible outcomes of an event are not equal, for instance, in an imbalanced situation, the concept of entropy can be applied to the information concerning the outcome. **Entropy** refers to the average amount of information obtained from an information source. The entropy of an information source *X* is denoted by *H*(*X*). Here, the information sources are the metrics in which the classifiers exhibit losses or gains as a result of the information entropy. These metrics include the specificity, F-score, recall, precision and G-mean. To determine *H*(*X*), we use

$$H\left(X\right)=\sum_{i=1}^{n}p_{i}log_{2}\left(\frac{1}{p_{i}}\right)$$ (23)

We can rewrite the above equation as

$$H\left(X\right)=p_{1}log_{2}\left(\frac{1}{p_{1}}\right)+\ldots p_{n}log_{2}\left(\frac{1}{p_{n}}\right)$$ (24)

where *p*_*i*_ is the probability of a single outcome with respect to the information source and *n* is the number of outcomes.

## 4 Experimental methodology and outcomes

The experiment was conducted on a computer with a 1.70 GHz Intel(R) Core(TM) i5-3317U CPU and 4 GB of RAM running 64-bit Windows 10. The accuracy of the learning algorithms was assessed using a statistical data management tool called Orange, version 2.7.

### 4.1 Data collection and preprocessing technique

In the current study, the ELFF open-source Java projects, which contain 289,132 methods, were used. The ELFF dataset is vailable at www.elff.org.uk/ESEM2016, [50]. Furthermore, these datasets contain a significant number of inconsistencies, necessitating proper preprocessing and cleaning to avoid misleading results.

#### 4.1.1 Feature selection procedure

To address the erroneous nature of the data applied in machine learning studies, accurate feature selection is important. First, we applied the derived optimal variables in data preprocessing as features that are correlated with the number of defects. Through these variables, the values of the defect density and the rate at which defects occur at the method level can be determined. We can deduce that the defect velocity determines the increase in the number of defects in software at the method level. Note that the terms “features”, “attributes” and “variables” are used interchangeably here as they refer to the same metrics. However, selecting the most relevant attributes enables the removal of irrelevant features from a dataset, thus simplifying data management. For this reason, feature selection is essential when preprocessing the datasets applied in machine learning studies for either regression or classification [51], [52].

Second, for the purpose of determining the average classifier performance, we applied a different feature selection technique. For each dataset, we determined the individual rank score of each feature, denoted by *rank*_*id*_, to enable us to identify the relevant features and ascertain their correlations with the target methods in the dataset, i.e., the defective methods. To achieve the above objective, we set an optimal threshold equal to the average rank score of all features, denoted by *Rank*_*ave*_. Features whose rank scores were equal to or greater than this threshold were selected, and those with lower rank scores were discarded. Algorithm 1 presents the steps of the feature selection procedure applied in the current study.

For this feature selection procedure, a filter-based selection module was selected. This module provides multiple feature scoring algorithms to ascertain the relationships between the independent variables (i.e., the features in the dataset) and the dependent variable (the target defective methods). Among the feature scoring methods offered by the module, the Pearson correlation method was selected to accurately determine the linear relationships between two variables (dependent and independent), which we expected to be relevant for identifying meaningful features. The Pearson correlation can also be used to quantify the strength of meaningful features in terms of correlation. The Pearson correlation *r* between two variables A and B can be computed as follows:

$$r=\frac{\text{cov}(A,B)}{\sigma_{a}\sigma_{b}}$$ (25)

where *cov* is the covariance,

*σ*_*a*_ is the standard deviation of A, and

*σ*_*b*_ is the standard deviation of B.

Based on the Pearson correlation, we can determine the individual rank score of each feature in a dataset.

The optimal threshold *Rank*_*ave*_ is calculated as

$$Rank_{ave}=\frac{1}{n}\sum_{i=1}^{n}d_{n}=\frac{1}{n}(rank_{id1}+rank_{id2}+\ldots rank_{idn})$$ (26)

where

*n* = the total number of features in the dataset,

*Rank*_*ave*_ = the average rank score, and

*rank*_*idi*_ = the rank score of the *i*-th individual feature.

**Algorithm 1** Steps of filter-based feature selection

1 **procedure** Steps of feature selection from each dataset

 **Input**: Datasets *DS* with universal feature set

 **Output**: Datasets with selected feature subset

2:  Analyze features in each dataset

3:  Record the total number of features in each dataset

4:  Apply feature selection module to each dataset

5:  Apply feature scoring method to rank each feature

6:  Record rank score of each feature in each dataset

7:  Determine *average* rank score of all features in each dataset

8  **for** *i* = 1 to *n* (each feature) **do**

9:   Set optimal value = average rank score (*Rank*_*ave*_)

10:   **if** *individual feature rank score rank*_*id*_ ≥ *Rank*_*ave*_ **then**

11:    Include feature in subset

12:   **else**

13:    Discard feature

14:    Record the total number of selected features for which *rank*_*id*_ ≥ *Rank*_*ave*_

15:   **end if**

16:  **end for**

17: **end procedure**

#### 4.1.2 Outlier removal from imbalanced method-level datasets

A dataset is imbalanced if the categories into which the data are to be classified are not almost equally represented [53]. Thus, data imbalance refers to the case in which the proportions of defective (minority) and defect-free (majority) modules or methods in a project are not equal. In addition to feature selection, an important phase of data preprocessing is the outlier removal. Therefore, we first checked whether the data contained outliers before performing further preprocessing tasks.

**Fig 3:**
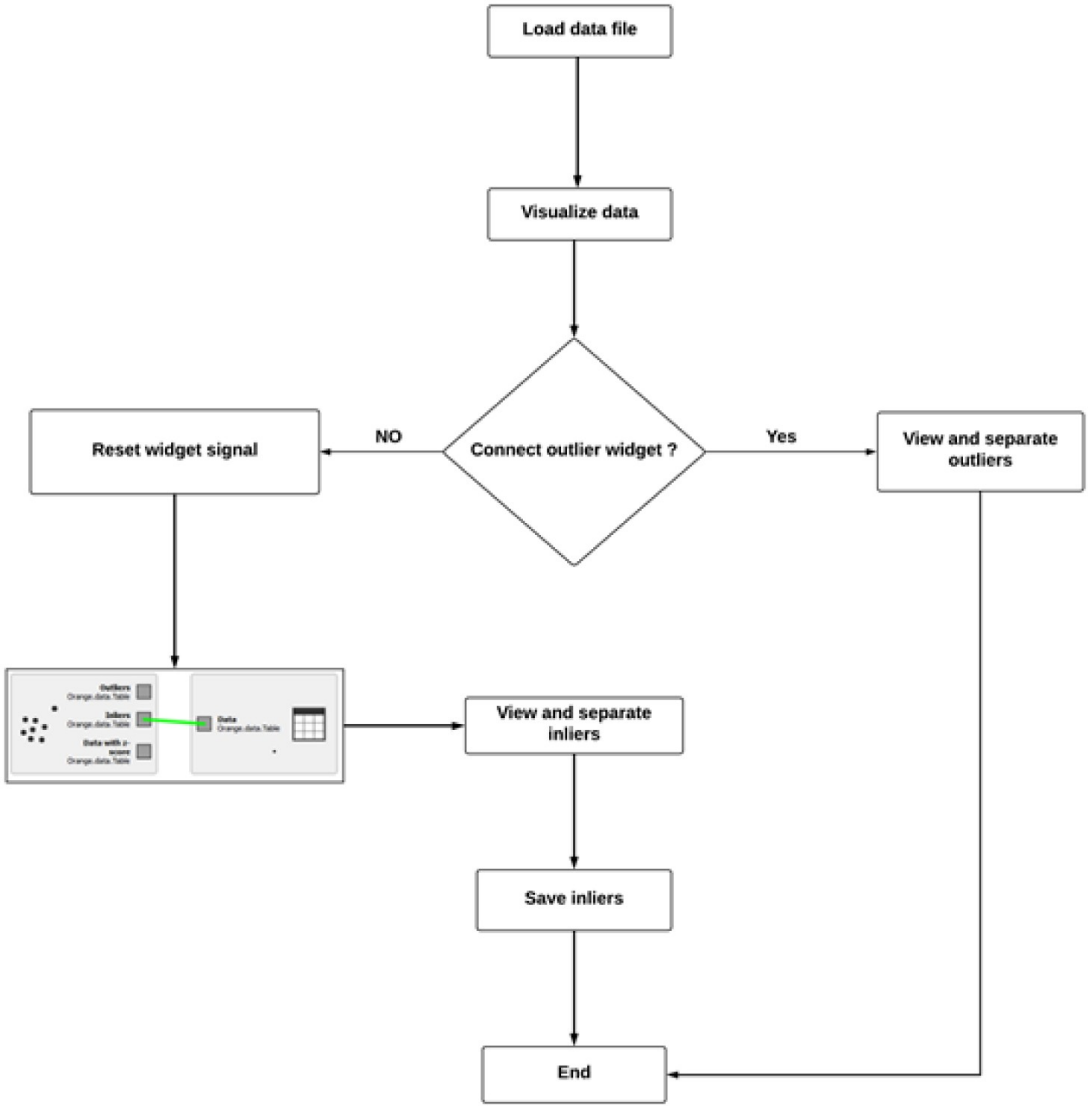
Outlier removal procedure.

Outliers are data points that are isolated from the main cluster(s) of data. Outliers may occur in datasets as a result of measurement variations or may be a manifestation of experimental error. In this study, outliers were identified and eliminated during the preprocessing phase to prevent them from impacting the results. Fig 3 illustrates how the outliers were identified and removed from the data. To identify outliers, first, we loaded the data file into the Orange workspace and visualized the inliers and outliers in the data. An outlier widget was then connected to the data file to enable the separation of the outliers. Thereafter, we reset the outlier widget signal to capture only the inliers in the data. To achieve a reliable, bias-free dataset that would be suitable for use in prediction, it was then necessary to subject the inliers to further preprocessing. The reason for removing outliers is to enable prediction models to learn only from the main clusters of data to ensure an accurate assessment of the models’ performance. If a prediction model is trained on data containing outliers, the resulting noise in the data can cause the model to produce misleading results.

**Fig 4:**
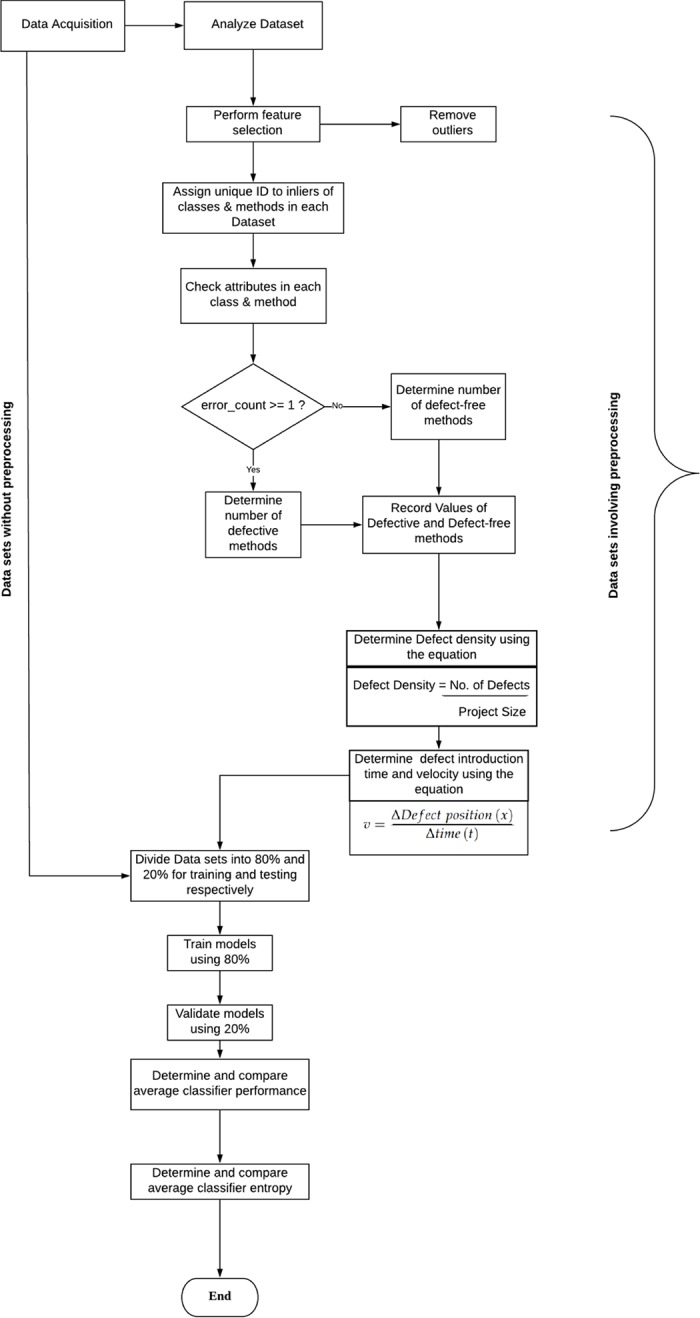
Flow diagram of data preprocessing to eliminate inconsistencies in the data.

Table 1 presents the detailed statistics of the preprocessed ELFF datasets at the method level. From left to right, the columns present the project name, the number of methods, the number of defective methods, the number of attributes, the percentage of defective methods, the corresponding method-level defect density, the time of defect occurrence and the corresponding defect velocity. Fig 4 presents a flow diagram of the data preprocessing steps applied to reduce data inconsistencies to ensure that the data applied in this study were free from bias. The data preprocessing technique is presented in Algorithm 2. During data preprocessing, we first analyzed the datasets applied in this study to understand their nature in detail. We determined the source, size, and format of each dataset. Second, we assigned a unique identifier to each dataset to enable us to accurately identify the defective methods in the datasets.

**Table 1:** Method-level statistics of the preprocessed ELFF datasets.

Project Name	No. of Methods	No. of Defective Methods	No. of Attributes	% Defective Methods	Defect Density (num/unit project size)	Defect Introduction Time (days)	Defect Velocity (num/day)
AutoPlot 2012	15781	191	33	1.2	0.0121	1615	19.50
Cdk1	9576	0	33	0	0	0	0
Cdk1.1	4276	73	33	1.7	0.0171	707	12.10
Cdk1.2	4366	0	33	0	0	0	0
Cmusphinx3.6	4819	15	33	0.30	0.0031	1763	5.50
Cmusphinx3.7	4826	10	33	0.20	0.0021	2144	4.50
Controltier3	6078	0	33	0	0	0	0
Controltier3.1	5799	52	33	0.9	0.0089	1142	10.20
Controltier3.2	4946	0	33	0	0	0	0
Drjava2008	15748	1012	33	6.40	0.0643	700	45.00
Drjava2009	3333	130	33	3.90	0.039	413	16.10
Drjava2010	4946	100	33	0.2	0.0020	700	14.1
Eclemma2	896	9	33	1.00	0.0100	423	4.20
Eclemma2.1	1081	37	33	3.40	0.0.0342	251	8.6
Ejit3	3357	48	33	1.43	0.0143	685	9.80
Genoviz5.4	1451	141	33	9.7	0.0972	173	16.80
Genoviz6	1269	117	33	9.20	0.0922	166	15.30
Genoviz6.1	4701	504	33	10.70	0.1072	292	31.70
Genoviz6.2	5704	210	33	3.70	0.0368	557	20.50
Genoviz6.3	8509	221	33	2.6	0.0259	811	20.90
HTMLUnit2008	4715	427	33	9.00	0.0906	323	29.20
HTMLUnit2009	1096	16	33	1.50	0.0146	387	5.70
HTMLUnit2010	7747	259	33	3.30	0.0334	681	22.70
JEdit5.2	5400	9	33	0.17	0.0017	2521	4.30
Jikesrvm2	4489	43	33	0.96	0.0096	967	9.30
Jikesrvm3	5113	149	33	2.90	0.0291	593	17.30
Jikesrvm3.1	3890	20	33	0.50	0.0051	1235	6.30
Jitterbit1.1	1155	22	33	1.90	0.019	349	6.60
Jitterbit1.2	11246	26	33	0.23	0.0023	3127	7.20
Jmol2	1347	38	33	2.80	0.0282	309	8.70
Jmol3	1402	35	33	2.50	0.024	336	8.40
Jmol4	1419	81	33	5.70	0.0571	223	12.70
Jmol5	89	4	33	4.50	0.0449	63	2.80
Jmol6	2170	280	33	12.90	0.129	183	23.70
Jmol7	2484	248	33	9.98	0.0998	223	22.30
Jmol8	1910	85	33	4.50	0.0445	293	13.00
Jmol9	3433	176	33	5.12	0.0513	366	18.80
Jmol10	3957	81	33	2.05	0.0205	621	12.70
Jmri2	4910	42	33	0.85	0.0086	1066	9.20
Jmri2.2	17011	175	33	1.03	0.0103	1817	18.70
Jmri2.4	11564	802	33	6.90	0.0694	577	40.10
Jmri2.6	2637	30	33	1.13	0.0114	680	7.80
Jppf4	2054	57	33	2.78	0.0278	384	10.70
Jppf4.1	2058	28	33	1.36	0.0136	550	7.50
Jppf4.2	298	5	33	1.67	0.0168	188	3.20
Jppf5	448	16	33	3.57	0.0357	158	5.70
Jppf5.1	3618	19	33	0.53	0.0053	1168	6.20
Jtds23072009	2005	27	33	1.34	0.0135	545	7.40
Jump1.5	5194	51	33	0.98	0.0098	1030	10.10
Jump1.6	3692	30	33	0.81	0.0081	955	7.70
Jump1.7	4064	26	33	0.64	0.0064	1127	7.20
Jump1.8	3090	13	33	0.42	0.0042	1213	5.10
Jump1.9	11661	201	33	1.72	0.0172	1164	20.00
OmegaT3.1	4347	35	33	0.81	0.0081	1036	8.40
OmegaT3.5	1812	30	33	1.66	0.0166	467	7.80
OmegaT3.6	2331	34	33	1.46	0.0146	565	8.30
Runawfe3.5	3282	0	33	0	0	0	0
Runawfe3.6	470	0	33	0	0	0	0
Runawfe4.1	1402	46	33	3.28	0.0328	292	9.60
Runawfe4.2	2136	0	33	0	0	0	0
Saros1.0.6	749	31	33	4.13	0.0414	190	7.90
Tango2008	3246	18	33	0.55	0.0055	1086	6.00
Unicore1.2	1756	67	33	3.80	0.0382	303	11.60
Unicore1.3	952	21	33	2.20	0.0221	294	6.50
Unicore1.4	2575	202	33	7.84	0.0784	256	20.10
Unicore1.5	4007	69	33	1.72	0.0172	683	11.70
Unicore1.6	2171	113	33	5.20	0.052	289	15.00
Xaware5	792	18	33	2.27	0.0227	264	6.00
Xaware5.1	6033	43	33	0.71	0.0071	1304	9.30
Xaware6	2843	0	33	0	0	0	0

When identifying the defective and defect-free methods, a binary decision (i.e., a choice between two alternatives) was made with an output of (0, 1) to ensure that accurate records were obtained during data preprocessing.

An error count greater than or equal to 1 indicated a defective method, whereas an error count of 0 indicated a defect-free method or module. This process was repeated for all datasets applied in the current study, as presented in Algorithm 2. In total, 80% of the preprocessed data were used as training data, and validation was performed using the remaining 20% of the data in both the regression and classification phases. This data ratio was selected to ensure that the learning algorithms would be adequately trained to avoid bias. Six selected classifiers were implemented on each dataset to determine their individual classification performances; thereafter, the average classification performance of each classifier on all datasets was determined accordingly. The total preprocessing time, which includes the load time, feature selection time, feature ranking time and outlier removal time, is presented in Table 2.

**Load time**: This is the time required to import the method-level data file into an Orange 2.7 data file. The load time is measured in seconds.

**Feature selection time**: This the time required to select all features from a dataset before ranking. The feature selection time is also measured in seconds.

**Feature ranking time**: This is the time consumed for ranking the features in the datasets, measured in seconds.

**Outlier removal time**: This is the time required to separate the outliers from each dataset.

**Table 2:** Preprocessing time for the method-level ELFF datasets.

Project Name	No. of Methods	No. of Defective Methods	File size (kB)	Load Time (sec)	Feature Selection Time (sec)	Feature Ranking Time (sec)	Outlier Removal Time (sec)	Total Preprocessing Time (sec)
AutoPlot 2012	15781	191	15,241	49.9	1.0	25.0	1.0	76.9
Cdk1	9576	0	14,184	46.4	1.0	25.0	1.0	73.4
Cdk1.1	4276	73	13,041	42.7	1.0	25.0	1.0	69.7
Cdk1.2	4366	0	12,107	39.6	1.0	25.0	1.0	66.6
Cmusphinx3.6	4819	15	3,476	11.4	1.0	25.0	1.0	38.4
Cmusphinx3.7	4826	10	3,492	11.4	1.0	25.0	1.0	38.4
Controltier3	6078	0	9,298	30.4	1.0	25.0	1.0	57.4
Controltier3.1	5799	52	9,297	30.4	1.0	25.0	1.0	57.4
Controltier3.2	4946	0	9,444	30.9	1.0	25.0	1.0	57.9
Drjava2008	15748	1012	11,356	37.2	1.0	25.0	1.0	64.2
Drjava2009	3333	130	13,246	43.4	1.0	25.0	1.0	70.4
Drjava2010	4946	100	14,668	48.0	1.0	25.0	1.0	75.0
Eclemma2	896	9	565	1.8	1.0	25.0	1.0	28.8
Eclemma2.1	1081	37	675	2.2	1.0	25.0	1.0	29.2
Ejit3	3357	48	20,096	65.8	1.0	25.0	1.0	92.8
Genoviz5.4	1451	141	7,340	24.0	1.0	25.0	1.0	51.0
Genoviz6	1269	117	7,151	23.4	1.0	25.0	1.0	50.4
Genoviz6.1	4701	504	6,951	22.8	1.0	25.0	1.0	49.8
Genoviz6.2	5704	210	7,383	24.2	1.0	25.0	1.0	51.2
Genoviz6.3	8509	221	7,782	25.5	1.0	25.0	1.0	52.5
HTMLUnit2008	4715	427	3,166	10.4	1.0	25.0	1.0	37.4
HTMLUnit2009	1096	16	7,521	24.6	1.0	25.0	1.0	51.6
HTMLUnit2010	7747	259	8,347	27.3	1.0	25.0	1.0	54.3
JEdit5.2	5400	9	6,233	20.4	1.0	25.0	1.0	47.4
Jikesrvm2	4489	43	8,757	28.7	1.0	25.0	1.0	55.7
Jikesrvm3	5113	149	14,284	46.8	1.0	25.0	1.0	73.8
Jikesrvm3.1	3890	20	15,069	49.3	1.0	25.0	1.0	76.3
Jitterbit1.1	1155	22	17,009	55.7	1.0	25.0	1.0	82.7
Jitterbit1.2	11246	26	31,932	105.0	1.0	25.0	1.0	132.0
Jmol2	1347	38	1,222	4.0	1.0	25.0	1.0	31.0
Jmol3	1402	35	1, 261	4.0	1.0	25.0	1.0	31.0
Jmol4	1419	81	1,309	4.0	1.0	25.0	1.0	31.0
Jmol5	89	4	1, 592	5.0	1.0	25.0	1.0	32.0
Jmol6	2170	280	1,975	6.5	1.0	25.0	1.0	33.5
Jmol7	2484	248	2,207	7.0	1.0	25.0	1.0	34.0
Jmol8	1910	85	2,818	9.0	1.0	25.0	1.0	36.0
Jmol9	3433	176	2,966	9.7	1.0	25.0	1.0	36.7
Jmol10	3957	81	4,244	13.9	1.0	25.0	1.0	40.9
Jmri2	4910	42	11,186	36.6	1.0	25.0	1.0	63.6
Jmri2.2	17011	175	13,787	45.0	1.0	25.0	1.0	72.0
Jmri2.4	11564	802	16,639	54.5	1.0	25.0	1.0	81.5
Jmri2.6	2637	30	18,911	62.0	1.0	25.0	1.0	89.0
Jppf4	2054	57	6,531	21.4	1.0	25.0	1.0	51.4
Jppf4.1	2058	28	6,607	21.6	1.0	25.0	1.0	48.6
Jppf4.2	298	5	6,700	21.9	1.0	25.0	1.0	48.9
Jppf5	448	16	6,421	21.0	1.0	25.0	1.0	48.0
Jppf5.1	3618	19	6,534	21.4	1.0	25.0	1.0	48.4
Jtds23072009	2005	27	2,814	9.2	1.0	25.0	1.0	36.2
Jump1.5	5194	51	10,823	35.4	1.0	25.0	1.0	62.4
Jump1.6	3692	30	11,661	38.0	1.0	25.0	1.0	65.0
Jump1.7	4064	26	12,206	40.0	1.0	25.0	1.0	67.0
Jump1.8	3090	13	12,380	40.5	1.0	25.0	1.0	67.5
Jump1.9	11661	201	12,880	42.1	1.0	25.0	1.0	69.1
OmegaT3.1	4347	35	4,087	13.4	1.0	25.0	1.0	40.4
OmegaT3.5	1812	30	4,707	15.4	1.0	25.0	1.0	42.4
OmegaT3.6	2331	34	5,095	16.7	1.0	25.0	1.0	43.7
Runawfe3.5	3282	0	27,752	90.8	1.0	25.0	1.0	117.8
Runawfe3.6	470	0	28,371	92.9	1.0	25.0	1.0	119.9
Runawfe4.1	1402	46	9,298	30.4	1.0	25.0	1.0	57.4
Runawfe4.2	2136	0	11,112	36.4	1.0	25.0	1.0	63.4
Saros1.0.6	749	31	1,198	3.9	1.0	25.0	1.0	30.9
Tango2008	3246	18	24,134	78.9	1.0	25.0	1.0	105.9
Unicore1.2	1756	67	1,630	5.3	1.0	25.0	1.0	32.3
Unicore1.3	952	21	1,882	6.2	1.0	25.0	1.0	33.2
Unicore1.4	2575	202	2,034	6.7	1.0	25.0	1.0	33.7
Unicore1.5	4007	69	3,094	10.1	1.0	25.0	1.0	37.1
Unicore1.6	2171	113	3,527	11.5	1.0	25.0	1.0	38.5
Xaware5	792	18	5,889	19.3	1.0	25.0	1.0	46.3
Xaware5.1	6033	43	6,867	22.5	1.0	25.0	1.0	49.5
Xaware6	2843	0	6,915	22.6	1.0	25.0	1.0	49.6

**Algorithm 2** Preprocessing technique for defect prediction

1: **procedure** Phases of Data Preprocessing for Predicting Possible Numbers of Defects and Average Classifier Entropy Evaluation

 **Input**: Datasets *DS*

 **Output**: Preprocessed data for determining the numbers of defects and average classifier performance ${\bar{P}}_{av}$ based on 10 × 10 cross-validation sampling in terms of both the number of folds and the number of repetitions of training/testing

2:  Analyze method-level datasets *DS*_*ml*_

3:  Perform feature selection on each dataset *DS*_*ml*_

4:  Remove outliers from each dataset *DS*_*ml*_

5:  **for** *i* = 1 to *n* **do**

6:   Assign unique IDs to the inliers of each *DS*_*ml*_

7:   Recheck features in each module of each dataset

8:   Remove incomplete and missing values

9:   Identify defective and defect-free modules *MD*_1−*n*_ among *DS*_*ml*_

10:   **for** *i* = 1 to *n* **do**

11:    **if** number of faults ≥ 1 **then**

12:     (*MD*) = 1

13:     *MD* = defective: *f*(number of faults present)

14:     Determine and record number and percentage of defects

15:    **else**

16:     (*MD*) = 0

17:     *MD* = defect-free: *f*(no faults present)

18:     Recheck feature completeness in *DS*_*ml*_

19:    **end if**

20:    **for** *i* = 1 to *n* **do**

21:     **First**, determine the average defect density $g=\frac{no.\;of\;defects}{project\;size}$

22:     Determine *v* and *t*; see Eq (2)

23:     Determine the correlations and record the impacts of *g*, *v* and *t* on the number of defects for the current version in datasets *DS*_*ml*_

24:     Estimate the possible number of defects in the new version

25:     **Second**, apply learning algorithms to the training data

26:     Evaluate the individual classifier performances *p* using the testing data

27:     Calculate the average classifier performance ${\bar{P}}_{av}=\frac{1}{n}\sum_{i=1}^{n}=\frac{1}{n}\left(p_{1}+p_{2}+\ldots p_{n}\right)$

28:  Evaluate the average classifier entropy performance *H*(*x*)

29:    **end for**

30:   **end for**

31:  **end for**

32: **end procedure**

### 4.2 Regression model for predicting the number of defects

A regression model was constructed for the purpose of estimating the possible number of defects in a new version of software. The prediction outcomes were then compared with the actual numbers of defects in the current versions.

$$Y=B_{0}+B_{1}X_{1}+B_{2}X_{2}+\;\varepsilon$$

In the above multiple regression equation, which contains two independent variables, *X*_1_ and *X*_2_, these two variables are treated as being independent of each other. However, multicollinearity will arise if *X*_1_ and *X*_2_ are correlated. In the above expression, *Y* represents the dependent variable and, as such, depends on *X*_1_ and *X*_2_. This indicates that *X*_1_ and *X*_2_ both contain relevant information about *Y*. If *X*_1_ and *X*_2_ are collinear, this situation can lead to redundancy, which will hinder the ability to generate accurate prediction outcomes. In such a situation, highly correlated metrics should be eliminated. When collinear variables are applied in a prediction model, the correlation among these supposedly independent variables tends to reduce the accuracy of the model [54]. To avoid the potential influence of collinearity on the prediction accuracy, we considered a simple regression model, which will accurately reflect the performance of the independent variable. *B*_0_ represents the intercept, while *B*_1_ and *B*_2_ are the coefficients of the independent variables *X*_1_ and *X*_2_. *ε* represents the standard error of our prediction.

We constructed a regression model with only one independent variable *X*_1_, i.e., the defect velocity, because it shows a firm but positive impact on the number of defects in terms of correlation. Based on an ANOVA of the defect velocity at the method level, the intercept and coefficient for the defect velocity were found to be approximately -96 and 17.7, respectively. These values were used to construct the regression equations. The regression equation below was applied to estimate the number of defects based on the above values:

$$Y=B_{0}+B_{1}X_{1}+\varepsilon$$ (27)

The resulting regression equation for estimating the number of defects at the method level is

$$Y=-96+17.7X_{1}+\varepsilon$$ (28)

**Example 1**. Suppose that a new version of the *Unicore* project, similar to an existing version, is to be developed. It is possible to estimate the number of defects in the new project at the method level using this regression equation. For instance, when developing a *Unicore*1.*n* version that is similar to the existing *Unicore*1.3 project, information on the existing project, such as the rate at which defects occurred in the current project (i.e., the defect velocity), can be helpful for predicting the defect characteristics of the future project. The *Unicore*1.3 project was characterized by a defect velocity of 6.50 defects per day. Therefore, the regression equation for estimating the possible number of defects in *Unicore*1.*n* at the method level is

Y=-96+17.7(6.50)+ε=19+ε (29)

This means that the predicted number of defects at the method level in the *Unicore*1.*n* version is 19 plus the standard error *ε*. For comparison, the actual number of defects in the *Unicore*1.3 project is 21. The reader is referred to Table 3 for details.

**Table 3:** Correlation coefficients between the predictor variables and the number of defects for the ELFF datasets at the method level.

Variable	Correlation Coefficient
Average defect introduction time	-4%
Average defect density	60%
Average defect velocity	93%

**Example 2**. Suppose that a new version of the *Jppf*5.1 project, similar to an existing version, is to be developed. It is possible to estimate the number of defects in the new project at the method level using this regression equation. For instance, when developing an *Jppf*5.*n* version that is similar to the *Jppf*5.1 project, information such as the defect velocity of the existing project can again be helpful for predicting the defect characteristics of the future version. The current version of the *Jppf*5.1 project was characterized by a defect velocity of 6.20 defects per day. Therefore, the resulting regression equation for estimating the possible number of defects in *Jppf*5.*n* at the method level is

Y=-96+17.7(6.20)+ε=14+ε (30)

This means that the predicted number of method-level defects in the *Jppf*5.1 version is 14 plus the standard error *ε*. The numbers of defects predicted as described above are compared with the actual numbers of defects in the results section.

**Example 3**. Suppose that a new version of the *Jitterbit*1.1 project, similar to an existing version, is to be developed. It is possible to estimate the number of defects in the new version at the method level using this regression equation. For instance, when developing a *Jitterbit*1.*n* version that is similar to the *Jitterbit*1.1 project, information such as the defect velocity of the existing project can be helpful for predicting the defect characteristics of the future project. The *Jitterbit*1.1 project was characterized by a defect velocity of 6.60 defects per day. Therefore, the resulting regression equation for estimating the possible number of defects in *Jitterbit*1.*n* at the method level is

Y=-6+17.7(6.60)+ε=21+ε (31)

This means that the predicted number of method-level defects in the *Jitterbit*1.*n* version is approximately 21 plus the standard error *ε*; by comparison, the number of defects in the current version is 22.

## 5 Results and discussion

In this section, we present the results and findings of the current study and the answers to our research questions.

**RQ1**. **How many studies have applied the aforementioned optimal variables when performing data preprocessing for defect prediction at the method level?**

Existing studies have focused on evaluating the performance of learning algorithms in binary defect prediction. Notably, only a few studies have considered the estimation of future defects in a new version of software, for instance, [23] and [3]. Of these 2 studies found, only the work reported in [3] shows that both the density and the rate at which defects occur can reveal essential information regarding the number of class-level defects revealed during data preprocessing and can be used to construct models to predict defects at the class level. However, these variables have not been implemented at the method level for either data preprocessing or predicting the number of defects. We therefore conclude that these variables have not been fully investigated as factors that impact the number of defects in software.

**RQ2**. **How do these variables address method-level data quality issues?**

Datasets are imbalanced by default and, as such, may be biased. In addition, the irrelevant and redundant features associated with existing datasets make them biased and erroneous. Therefore, there is a need for a white-box approach for properly cleaning existing datasets, especially at the internal stage for method-level studies. In the current study, the defect density reflects the intensity of defects at the method level, whereas the defect velocity and time reflect the rate at which these defects occur. Such information makes it possible to investigate the impacts of these variables on the number of defects. The results therefore suggest that the quality of a new software version can be improved by reducing or eliminating the defects estimated to occur in the near future and by offering a means of properly cleaning datasets.

**RQ3**. **What are the limitations of the current research in the supervised machine learning domain?**

**Fig 5:**
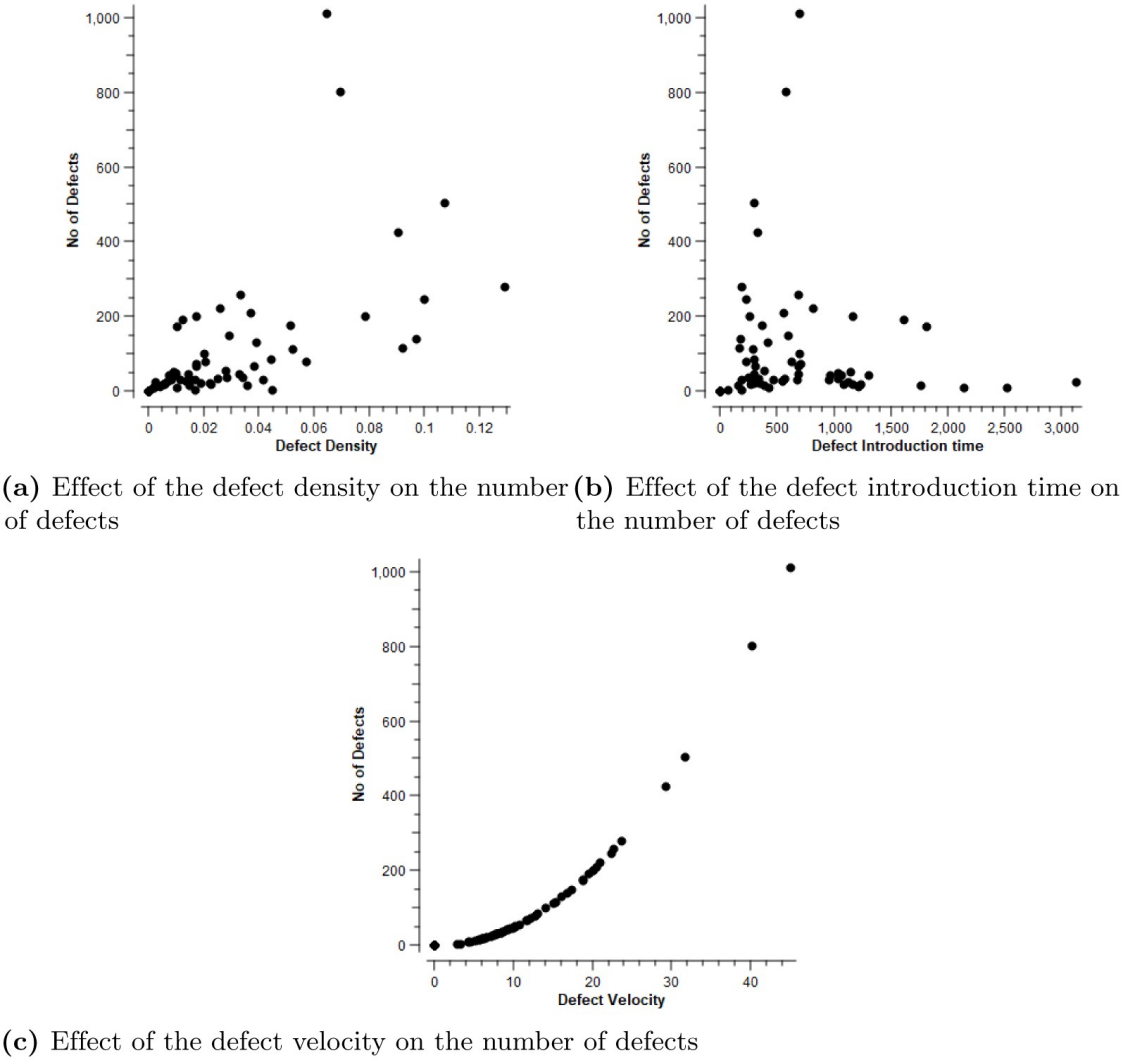
Graphical illustration of the impact of the defect density, defect introduction time and defect velocity on the number of defects at the method level. (a) Effect of the defect density on the number of defects, (b) Effect of the defect introduction time on the number of defects, (c) Effect of the defect velocity on the number of defects.

Regarding the limitations of the current research, we focused on one specific factor, which forms the basis of our **RQ3.1**: Is there evidence that these optimal variables have not been fully applied in machine learning studies when attempting to predict the number of defects in a new version of software at the method level? Numerous studies have considered various variables while improving the performance of learning models. However, the current study provides sufficient evidence that studies applying the variables considered here (defect density, velocity and time) at the method level for both data preprocessing and defect prediction for new software versions are lacking. The method-level statistics of the preprocessed ELFF datasets are presented in Tables 1 and 2, respectively. In response to the stated hypotheses in the current study, it is confirmed that the defect density at the method level of software is influenced by the number of defects, since the software defect density increases with an increase in the number of defects and decreases when the number of defects is reduced. The results obtained in this study further confirm that the ratio of defect velocity over defect introduction time is equivalent to the defect density at the method level. In addition, a software program with a high number of defects will be associated with a high but equal defect density and rate (i.e., the ratio of defect velocity and time) at the method level. Table 1 presents the method-level statistics of the preprocessed ELFF datasets with the corresponding results for the defect density, velocity and time. The results for the correlation coefficients of the derived variables are presented in Table 3: the defect introduction time was found to have a correlation coefficient of -4%, indicating a negative correlation with the number of defects; the defect density had a correlation coefficient of 60%, indicating a moderate positive correlation with the number of defects; and the average defect velocity achieved a correlation coefficient of 98%, showing a considerable, firm and positive correlation with the number of defects. Fig 5(a), 5(b) and 5(c) presents a graphical illustration of the impacts of the defect density, defect introduction time and defect velocity on the number of defects as found at the method level. In each plot in these figures, the number of defects is plotted on the *y* axis, whereas the corresponding predictor variable is plotted on the *x* axis, with the defect velocity showing an exponential relationship with the number of defects.

Table 4 presents the results of defect prediction at the method level for the ELFF datasets. From left to right, the columns show the project name, the number of methods, the number of defects present in the current version, the predicted number of defects in the new version and the standard error of our prediction in the form of a percentage. The method-level prediction errors are less than 30%, implying that the accuracy of the results at the method level is greater than 70%. Note that in some cases, the predicted values are negative, implying that the number of predicted defects is less than 0; in such a scenario, we assume that the predicted number of defects is actually 0. For instance, if the predicted number of defects has a negative value, i.e., is below 0, the corresponding value is reported as 0. In addition, prediction results are not presented for all datasets because some of the datasets were used as the validation set. To satisfy the objectives of the current study, we compared the results obtained by training several learning algorithms using the raw and cleaned datasets. The results obtained after preprocessing show that the errors present in the datasets can hinder the proper assessment of the average performance of the learning algorithms. These errors contribute to the level of uncertainty in the datasets. As seen from a comparison of the average classification performances of the six selected classification models on the ELFF datasets before and after preprocessing, as presented in Tables 5 and 6. After preprocessing of the ELFF datasets, both the LR and KNN classifiers achieved the same CA of 98.57%. The RF model achieved an average CA of 97.14%, while the naïve Bayes and neural network models achieved CA values of 95.71% and 94.29%, respectively. Notably, the LR and KNN models appeared to tie in terms of the recall, specificity, F-score, MCC, J-coefficient and G-mean, with average results of 93.75%, 96.26%, 92.78%, 87.50% and 96.37%, respectively. The naïve Bayes classifier achieved the best performance in terms of the AUC, with an average value of 99.39%. The AUC captures all performance-related information and allows the essential links related to classifier performance to be clearly observed [55]. The KNN classifier achieved the best results in terms of the Brier score and information score, with values of 3.66% and 39.70%, respectively. Notably, the results of this study are significant compared with the results reported in [9].

**Table 4:** Comparison between the actual and predicted numbers of defects at the method level in the ELFF datasets and the corresponding percentage errors.

Project Name	No. of Methods	No. of Defects in Current Version	Predicted No. of Defects in New Version	Percentage Error
Cdk1	9576	0	0	0%
Cdk1.2	4366	0	0	0%
Controltier3	6078	0	0	0%
Controltier3.2	4946	0	0	0%
Genoviz6.1	4701	504	466	8%
HtmlUnit2008	4715	427	422	1.2%
HtmlUnit2010	7747	259	307	19%
Jitterbit1.1	1155	22	21	5%
Jitterbit1.2	11246	26	31	19%
Jmol6	2170	280	325	16%
Jmol7	2484	248	300	21%
Jppf5.1	3618	19	14	26%
Jump1.7	4064	26	32	23%
Jikesrvm3.1	3890	20	16	20%
Runawfe3.5	3282	0	0	0%
Runawfe3.6	470	0	0	0%
Runawfe4.2	2136	0	0	0%
Unicore1.3	952	21	19	10%
Xaware6	2843	0	0	0%

**Table 5:** Average classifier performance on the ELFF datasets before data preprocessing.

Classifier	CA	Sens	Spec	AUC	F-score	Prec	Recall	Brier	MCC	J-coef	IS	G-mean
Naïve Bayes	0.8551	0.8541	0.5267	0.5602	0.9148	0.9932	0.8541	0.2803	0.1714	0.3808	-0.5890	0.9210
LR	0.9755	0.9992	0.0203	0.5402	0.9876	0.9763	0.9992	0.0442	0.0583	0.0195	-0.0405	0.9877
Neural network	0.9980	0.9994	0.6235	0.6663	0.9989	0.9986	0.9994	0.0029	0.6338	0.6229	0.1355	0.9989
KNN	0.9791	0.9965	0.1919	0.5443	0.9892	0.9822	0.9965	0.0346	0.2849	0.1915	0.0002	0.9893
SVM	0.9754	0.9996	0.0058	0.3769	0.9875	0.9758	0.9996	0.0469	0.0255	0.0054	-0.0146	0.9876
RF	0.9972	0.9992	0.6053	0.6665	0.9986	0.9979	0.9992	0.0050	0.6225	0.6045	0.0865	0.9985

**Table 6:** Average classifier performance on the ELFF datasets after data preprocessing.

Classifier	CA	Sens	Spec	AUC	F-score	Prec	Recall	Brier	MCC	J-coef	IS	G-mean
Naïve Bayes	0.9571	0.9754	0.9754	0.9939	0.9085	0.8637	0.9754	0.0856	0.8316	0.9508	0.3538	0.9139
LR	0.9857	0.9375	0.9375	0.9672	0.9626	0.9919	0.9375	0.0761	0.9278	0.8750	0.1174	0.9637
Neural network	0.9429	0.7500	0.7500	0.8627	0.8175	0.9623	0.7500	0.1071	0.6850	0.5000	0.1871	0.8380
KNN	0.9857	0.9375	0.9375	0.9334	0.9626	0.9919	0.9375	0.0366	0.9278	0.8750	0.3970	0.9637
SVM	0.9405	0.9129	0.9129	0.9918	0.8723	0.8416	0.9129	0.0590	0.7511	0.8258	0.2389	0.8747
RF	0.9714	0.8750	0.8750	0.9805	0.9205	0.9842	0.8750	0.0505	0.8522	0.7500	0.3215	0.9250

The classifier performance results obtained after data preprocessing are noteworthy. In terms of the level of uncertainty in each dataset, which is defined as the average amount of information regarding the uncertainties of the learning outcomes as assessed using the evaluation metric, the results indicate that the outcomes of the learning algorithms exhibit uncertainties characterized by the inconsistencies in the imbalanced datasets at the method level, as presented in Table 7. In summary, the present study shows that incorrect preprocessing of a dataset can generate misleading results in a defect prediction study. Moreover, there is a possibility that classifiers may lose their performance capabilities when impurities, such as inconsistencies and uncertainties, are present in a software dataset at the method level. The average levels of uncertainty are presented in terms of the information entropy in *bits* shown in Table 7. Higher information entropy values indicate higher uncertainties in classification, whereas lower information entropy values suggest lower uncertainties regarding the accuracy of the obtained results.

**Table 7:** Average classifier information entropy in *bits* at the class level in the ELFF datasets.

Classifier	CA	Sens	Spec	AUC	F-score	Prec	Recall	Brier	MCC	J-coef	IS	G-mean
Naïve Bayes	≈ 0.24	≈ 0.16	≈ 0.16	≈ 0.05	≈ 0.14	≈ 0.56	≈ 0.16	≈ 0.41	≈ 0.64	≈ 0.13	≈ 0.90	≈ 0.41
LR	≈ 0.10	≈ 0.33	≈ 0.33	≈ 0.20	≈ 0.23	≈ 0.07	≈ 0.33	≈ 0.38	≈ 0.36	≈ 0.54	≈ 0.51	≈ 0.22
Neural network	≈ 0.31	≈ 0.80	≈ 0.80	≈ 0.57	≈ 0.68	≈ 0.23	≈ 0.80	≈ 0.47	≈ 0.92	≈ 1.00	≈ 0.66	≈ 0.62
KNN	≈ 0.10	≈ 0.33	≈ 0.33	≈ 0.35	≈ 0.23	≈ 0.07	≈ 0.33	≈ 0.22	≈ 0.37	≈ 0.54	≈ 0.95	≈ 0.22
SVM	≈ 0.32	≈ 0.42	≈ 0.42	≈ 0.68	≈ 0.55	≈ 0.62	≈ 0.42	≈ 0.32	≈ 0.78	≈ 0.65	≈ 0.78	≈ 0.54
RF	≈ 0.18	≈ 0.54	≈ 0.54	≈ 0.13	≈ 0.39	≈ 0.12	≈ 0.54	≈ 0.08	≈ 0.60	≈ 0.80	≈ 0.90	≈ 0.38

## 6 Conclusions

Many organizations have been greatly affected by financial loss as a result of software defects. For instance, in 2004 and 2005, software defects caused the UK Inland Revenue to issue tax-credit overpayments amounting to $3.45 billion. Between 2003 and 2004, the AT&T wireless service faced software defect problems that led to a revenue loss of $100 million. The United States Internal Revenue Service suffered an enormous loss of $4 billion in 1997 as a result of software defects. Furthermore, in 1994, the United States Federal Aviation Administration faced a loss of $2.6 billion linked to its advanced automation system. Also in 1994, Chemical Bank customers suffered a total loss of $15 million to their bank accounts as a result of software error [56]. Such problems can be avoided by applying an effective defect prediction approach to improve the quality of software products, thereby protecting organizations from financial losses and safeguarding the future of their businesses [56].

Software experts agree that software failures occur on a regular basis, and it is also very obvious that the causes of such failures can be predicted and/or avoided. Sadly, however, many organizations do not agree that such preventative measures require urgent attention even though, if neglected, software defects can cause significant harm [56]. Considering that software failures can have enormous negative impacts on both businesses and society, it is critical to propose some suitable means of addressing these problems, from which both software companies and society will benefit [24], [35], [36]. In the past, researchers have proposed various approaches to address software failures; however, previous studies have not fully addressed the issue of software defects. Therefore, more research is still required to investigate factors that influence the number of defects as well as to discover means of predicting the number of defects at the method level in a new software product as a means of preventing software failure. In the current study, we have shown that the derived variables proposed previously [3] for use at the class level can be extended to the method level for both data preprocessing and forecasting the number of defects in a future software version, which we believe will provide software teams with helpful information during software testing. We have also shown that various learning algorithms can yield accurate results when applied to data preprocessed using the presented approach. Moreover, the results reported in the current study further show that erroneous datasets can led to misleading research findings, and their use must therefore be avoided. In our future work, we hope to further investigate the factors that may impact the number of defects at the method level in a new software version, and we call on other researchers to follow suit.

## Supporting information

S1 Datasets (RAR)
